# Coated trace minerals improved carcass characteristics and meat quality in growing sheep in association with enhanced antioxidant status and shifts in the rumen microbiota

**DOI:** 10.3389/fmicb.2026.1942183

**Published:** 2026-08-25

**Authors:** Shoupei Zhao, Xiaoqi Zhao, Yifan Ren, Jia Zhou, Xiaojun Ni, Mingyu Cao, Bai Xue, Guobo Quan

**Affiliations:** 1 Animal Nutrition Institute, Sichuan Agricultural University, Chengdu, China; 2 Yunnan Animal Science and Veterinary Institute, Kunming, China; 3 Chongqing Academy of Animal Sciences, Chongqing, China

**Keywords:** sheep production, mineral supplementation, meat quality, rumen fermentation, immune function

## Abstract

**Introduction:**

Trace minerals (TM) are essential for maintaining normal rumen microbial function; however, excessive levels may exert toxic effects on rumen microorganisms and impair fiber digestion. This study aimed to evaluate the effects of coated trace minerals (CTM) on rumen fermentation, immune function, antioxidant status, slaughter performance, and meat quality in sheep, and to identify key differential rumen bacteria, thereby elucidating mechanisms underlying trace mineral regulation of rumen homeostasis and host responses.

**Methods:**

Thirty Yunnan fine-wool male sheep (4 months of age; 29.7 ± 0.3 kg) were randomly assigned to a control group (CON), an inorganic TM group (ITM), and a CTM group, with a 10-day adaptation and a 30-day feeding trial.

**Results:**

Liver concentrations of Fe, Cu, Mn, and Zn increased in both supplemented groups compared with CON (*p* < 0.05), with greater accumulation observed in CTM (*p* < 0.05). Both ITM and CTM improved carcass characteristics, reflected in increased slaughter rate (*p* < 0.05) and half carcass meat rate (0.05 < *p* < 0.1), with CTM showing additional benefits in meat quality, including a greater reduction in shear force (*p* < 0.05). Immune parameters, including IgA, IgG, IgM, C3, and C4, were significantly enhanced (*p* < 0.05), especially in the CTM group (*p* < 0.05). Antioxidant capacity was also improved, with increased catalase activity and decreased malondialdehyde levels (*p* < 0.05). Rumen fermentation was enhanced by TM supplementation, as shown by increased microbial protein and total volatile fatty acids, including acetate, butyrate, and isovaleric acid (*p* < 0.05), while rumen pH tended to decrease and NH₃-N tended to increase (0.05 < *p* < 0.1). No significant differences were observed in alpha diversity indices among groups (*p* > 0.05). However, CTM selectively enriched *Ruminococcus*, *Selenomonas 1*, and *Prevotella* while reducing *Christensenellaceae R-7 group* and *Bradyrhizobium* abundance (*p* < 0.05), suggesting a microbial shift potentially favorable for fiber degradation. Correlation analysis explored potential relationships between rumen microbiota and phenotypic indicators.

**Conclusion:**

In conclusion, CTM more effectively modulate rumen microbial composition, enhance immune and antioxidant capacity, and improve carcass characteristics and meat quality compared with ITM, indicating that CTM represent a more efficient strategy for optimizing rumen function and production performance in sheep.

## 1 Introduction

Trace minerals (TM) are essential nutrients required in small quantities for the proper functioning of animals and the maintenance of vital physiological processes (Brady et al., 2025; Hurlbert et al., 2024). As cofactors of metalloenzymes, TM are essential for various biological processes, including regulating oxidative balance, synthesizing vitamins and proteins, and supporting immune cell function (Andrieu, 2008; Goff, 2018). Previous studies have shown that a deficiency in TM can impair production and reproductive performance (Spears et al., 2022; Van Emon et al., 2020). Due to the considerable variation in the concentration and bioavailability of plant-based feeds, which are often insufficient to meet the needs of animals, feeding recommendations include iron (Fe), zinc (Zn), copper (Cu), manganese (Mn), iodine (I), and selenium (Se) (Brugger et al., 2022). For ruminant species, it is also recommended that cobalt (Co) fulfills the specific needs of the rumen microbiota and promote the synthesis of rumen cobalamin (NASEM 2016).

Currently, TM are commonly classified into inorganic and organic forms. Inorganic TM (ITM) are the primary forms supplemented in livestock and poultry feed. These forms are widely available and represent a cost-effective method of supplementation. However, ionic bonds often dissociate during the passage through the gastrointestinal tract, allowing for interactions with other molecules and thereby reducing the bioavailability of TM (Spears, 2003). Moreover, in the components of premixed feed, TM generally exhibits characteristics such as moisture absorption, caking, and difficulty in crushing (Lu et al., 2020). ITM also possess strong redox capabilities, which can accelerate the oxidation of sensitive ingredients (such as vitamins and fats) in feed, leading to losses, increased production costs, and reduced animal performance (Santos et al., 2015). In recent years, organic TM (OTM) have demonstrated superior efficacy; however, their inclusion in poultry diets may increase costs to approximately four times those of ITM (Lu et al., 2020). Previous studies have indicated that, due to the mutual antagonism and synergy among TM, as well as their complex interactions, research on single elements is often insufficient to meet the body’s normal physiological requirements. In contrast, supplementation with compound TM has been shown to produce more favorable outcomes than single-element supplementation, likely because of reduced interactions and competition during mineral absorption (Liu et al., 2016). This is particularly important in ruminants with complex rumen structures, where coating technologies enable a greater proportion of TM to bypass ruminal interference and reach the intestine for effective absorption and utilization. Moreover, compared with OTM, such strategies are associated with lower feed costs.

TM are essential for the normal functioning of rumen microorganisms (Durand and Kawashima, 1980). However, excessive amounts of certain TM can have toxic effects on rumen microorganisms and impair fiber digestibility (Mion et al., 2022). The bioavailability and efficiency of technical parameters relative to ruminants may be largely influenced by rumen solubility and the impact on the rumen environment (Vigh et al., 2023). Therefore, this phenomenon may be associated with the excessive dissociation of ITM in the rumen, which can negatively affect digestive processes and fermentation, thereby potentially impairing overall animal performance. Supplementing with CTM appears to provide sufficient TM for utilization by rumen microbiota, benefiting both fiber digestion and rumen fermentation. Some studies suggest that changes in the rumen environment may be caused by alterations in the rumen microbiome; however, there is currently a lack of relevant research (Pino and Heinrichs, 2016). Currently, most studies have primarily focused on improving animal production performance through the application of CTM, whereas limited attention has been given to their effects on slaughter performance (e.g., dressing percentage and meat quality) and rumen fermentation, particularly in comparison with monogastric animals. The mechanisms underlying the observed reductions in fiber degradation rates remain unclear. Regulation of the rumen microbiota, thereby enhancing nutrient absorption, may ultimately influence muscle fiber characteristics and intramuscular fat deposition, which are critical determinants in the improvement of meat quality (Li et al., 2024; Zhang et al., 2026). Therefore, our aim is to evaluate the effects of adding CTM on rumen fermentation and to identify key differential bacteria that undergo significant changes in the rumen microbiome, thereby explaining the mechanisms through which TM affect the ruminal internal environment of ruminants. Simultaneously, we investigated the effects of coated TM on immunity, slaughter performance, and meat quality, with the goal of optimizing the utilization of TM in ruminants. It was hypothesized that encapsulated trace minerals could reduce excessive ruminal mineral release, enhance intestinal mineral absorption and utilization efficiency, and modulate the rumen microbial composition, thereby improving antioxidant status, immune function, carcass characteristics, and meat quality in sheep.

## 2 Materials and methods

All experimental procedures involving animals adhered to the Chinese Guidelines for Animal Welfare and received approval from the Ethics Committee of the Experimental Animal Committee of Animal Nutrition Institute, Sichuan Agricultural University (Approval number: #SCAUAC2019-36).

### 2.1 Animal, housing, and experimental design

The experiment was carried out at Kunming Yixingheng Animal Husbandry Technology Co., Ltd. (Kunming, China) from August to September for 40 days, including 30 d experimental period with 10 d of adaptation. A total of 30 Yunnan semi-fine wool male sheep (*Ovis aries*) of 4 months and BW at 29.7 ± 0.3 kg were randomly divided into 3 groups (*n* = 10), with each animal serving as an individual experimental replicate. There was no difference in the initial BW among groups (*p* > 0.05). The treatments included: (1) a basal diet without additional TM supplementation (CON); (2) a basal diet supplemented with ITM (ITM), which consisted of ferrous sulfate heptahydrate, copper sulfate pentahydrate, manganese sulfate monohydrate, zinc sulfate heptahydrate, sodium selenite pentahydrate, potassium iodide, and cobalt chloride hexahydrate; and (3) a basal diet supplemented with 400 mg/kg CTM (CTM), which contained Fe, Zn, Mn, Cu, I, Co, and Se at 120, 85, 50, 16, 1.0, 0.8, and 0.8 mg/g, respectively. The coating was prepared using a composite coating of resistant starch, maltodextrin, and carboxymethyl cellulose via liquid–solid phase fusion. The ruminal bypass rate of CTM was previously determined to be 65.87% using the nylon bag technique in rumen-cannulated sheep, as reported in our previous study (Zhou et al., 2022). The ITM and CTM supplements provided identical amounts of TM. The supplementation level of CTM was determined from a preliminary dose–response (200, 400, 600, and 800 mg/kg of diet) trial, in which 400 mg/kg was identified as the optimal dietary level for growing Yunnan semi-fine wool sheep based on growth performance and immune function (Ren et al., 2023). The composition and nutrient levels of basal diet are shown in Table 1. During the experimental period, each sheep was housed individually and fed twice daily at 0700 and 1700 h. Water was provided ad libitum. CTM was thoroughly mixed with the concentrate before feeding.

**Table 1:** Composition and nutrient levels of basal diets (DM basis) %.

Items	Content
Ingredients/%	
Corn	36
Soybean meal	7.8
Wheat bran	5.1
NaCl	0.22
NaHCO_3_	0.38
Premix^1^	0.5
Corn silage	30
Wheat straw	20
Total	100.00
Nutrient levels^2^	
Metabolic energy/MJ·kg^−1^	9.36
Crude protein	10.52
Neutral detergent fiber	36.72
Acid detergent fiber	18.91
Calcium	0.36
Phosphorus	0.33
Iron	84.61
Zinc	25.47
Copper	18.65
Manganese	5.23
Iodine	0.09
Cobalt	0.05
Selenium	0.02

^1^The premix provides VA 10,000 IU, VD3 1,000 IU, VE 50 IU per kilogram. ^2^Metabolic energy was calculated, while other indicators were measured.

Four sheep with similar body condition and body weight were randomly selected from each group for slaughter on the d 30 of the experiment, and feeding was stopped at 16 h before slaughter.

### 2.2 Blood collection and serum biochemical analysis

Blood samples (10 mL) were collected from the selected sheep via jugular vein into tubes without anticoagulant on the morning of day 30 of the trial period. Serum was separated by centrifugation at 4 °C and 1,600 × g for 10 min, and stored at −20 °C for the future analysis.

The antioxidant indicator (superoxide dismutase (SOD), catalase (CAT), and malondialdehyde (MDA)) were measured according to the operating procedures of the kit (Nanjing Jiancheng Bioengineering Institute, Nanjing, China). And the immunity indices (immunoglobulin A (IgA), immunoglobulin M (IgM), immunoglobulin G (IgG), complement component C3 (C3), complement component C4 (C4), and endotoxin (ET)) were assayed according to the instructions of ELISA reagent kit (Jiangsu Enzyme Free Industry Co., Ltd., Jiangsu, China).

### 2.3 Carcass characteristics measurements

The slaughtering process followed the operating procedures of the national standard (GB/T 43562-2023, Sheep and Goat Slaughter, China). The digestive tract and red viscera (heart, liver, spleen, lungs, kidneys, testes) were dissected, ligated, and weighed after fat removal. Hot carcass weight was recorded 1 h postmortem. The left half of the carcass was dissected into meat and bone, and their weights were recorded. Slaughter rate, half-carcass net meat rate, and meat-to-bone ratio were calculated. The slaughtering rate, half carcass net meat rate and half carcass meat to bone ratio were calculated as follows:

Slaughter rate=carcass weight/fasting live weight×100%

$$\begin{array}{l} \text{Half carcass}\;\mathrm{net}\;\text{meat rate}=\text{Half Carcass}\;\mathrm{Net}\;\text{Meat Weight}/\\ \text{Half Carcass Body Weight}\times \\ 100\% \end{array}$$

$$\begin{array}{l} \text{Half carcass meat to bone ratio}=\text{Half Carcass}\;\mathrm{Net}\;\text{Meat Weight}/\\ \text{Half Carcass}\;\mathrm{Net}\;\text{Bone Weight}\times \\ 100\% \end{array}$$

### 2.4 Liver sample collection and trace minerals deposition analysis

A small sample from the lower left lobe of the liver was collected, rinsed with physiological saline to remove residual blood, minced, aliquoted into three tubes, and stored at −80 °C for the analysis of hepatic trace mineral concentrations.

TM concentrations in liver samples were determined by inductively coupled plasma mass spectrometry (ICP–MS; iCAP™ RQ ICP-MS, Thermo Fisher Scientific, Waltham, MA, USA) following microwave-assisted digestion, in accordance with the Chinese National Standard GB 5009.268–2016. Calibration standards containing the minerals of interest were obtained from the TMRM Quality Control Standard Material Center (Changzhou, China) and diluted to appropriate concentrations with the sample diluent prior to analysis.

### 2.5 Meat samples collection and meat quality analysis

A total of 500 g longissimus dorsi muscle was excised for measurement of pH, color, cooking loss, water holding capacity, and shear force.

The pH of the longissimus dorsi muscle was determined at 45 min and 24 h postmortem using a portable meat pH meter (pH-STAR, Matthäus, Germany). Prior to measurement, the instrument was calibrated using standard buffer solutions of pH 4.01 and pH 7.00 according to the manufacturer’s instructions, with automatic temperature compensation. The electrode probe was inserted approximately 2 cm into the muscle at each measurement site. Three measurements were taken per sample, and the average value was used for statistical analysis.

Meat color parameters, including lightness (L*), redness (a*), and yellowness (b*), were measured after allowing the muscle surface to bloom for 45 min at 4 °C. Surface fascia and visible fat were carefully removed before measurement. Color was determined using a colorimeter (CR-400/410, Konica Minolta, Osaka, Japan) equipped with illuminant C, an 8-mm aperture, and a 2° standard observer angle. The instrument was standardized against a white calibration plate before each measurement session. Three readings were obtained from different locations on each sample, and the mean value was recorded.

Drip loss was measured following a suspension method. Fresh muscle samples were weighed (W_1_), suspended vertically by a hook inside an inflated plastic bag to avoid contact with the bag surface, and stored at 4 °C for 24 h. After storage, samples were removed, gently blotted with filter paper to remove surface moisture, and reweighed (W_2_). Drip loss was calculated using the following equation:

$$\text{Drip loss}\;(\%)=[(W_{1}-W_{2})/W_{1}]\times 100$$

Cooking loss was determined using muscle samples cut into cuboids measuring 2.5 cm × 2 cm × 2 cm. Individual samples were weighed before cooking (W_1_), placed in polyethylene bags, and heated in an 80 °C water bath until the geometric center temperature reached 70 °C, as monitored using a calibrated thermometer. After reaching the target temperature, the samples were removed from the water bath and allowed to cool at room temperature for 60 min. Surface moisture was gently removed using filter paper, and the cooked samples were reweighed (W_2_). Cooking loss was calculated using the following equation:

$$\text{Cooking loss}\;(\%)=[(W_{1}-W_{2})/W_{1}]\times 100$$

Three replicate samples were analyzed per animal.

Shear force was measured using the cooked samples obtained from the cooking loss determination. Subsequently, each cooked sample was cut into cubes measuring 1 cm × 1 cm × 1 cm. The shear force was determined using the Texture Analyzer (XT2, Stable Micro Systems Ltd., Godalming, Surrey, UK). The measurement was performed perpendicular to the muscle fiber direction of each sample. Three different positions were measured for each cube, and the average value was recorded as the shear force of the sample. Three replicate samples were analyzed per animal, and each sample was measured three times, with the mean value used for statistical analysis.

### 2.6 Rumen fluid sample collection and rumen fermentation analysis

Rumen fluid was rapidly collected after rumen incision and filtered through four layers of sterile gauze. The filtrate was dispensed into 10 mL cryogenic tubes (10 tubes in total) and immediately stored in liquid nitrogen for subsequent determination of rumen fermentation parameters.

The pH of the rumen fluid was recorded on-site with a portable pH meter (DLX-PH-05–1, Delixi Electric, China). Ammonia nitrogen (NH_3_-N) was determined by the colorimetric method using a spectrophotometer (UV-3600i Plus, Shimadzu, Japan) (Weatherburn, 1967). Volatile fatty acids (VFA) were measured by gas chromatography using a Varian CP-3800 gas chromatograph (Agilent Technologies, Santa Clara, USA) (Erwin et al., 1961), and the acetic acid to propionic acid ratio (A/P) was calculated. The microbial protein (MCP) concentration in rumen fluid was determined using the bicinchoninic acid protein quantification kit (Nanjing Jiancheng Bioengineering) (Berthiaume et al., 2010). The MCP was separated by using differential centrifugation and cracked and precipitated by adding the cracking solution.

### 2.7 Rumen bacterial communities

DNA extraction. The rumen fluid DNA was extracted using ZymoBIOMICS TM DNA Microprep Kit (Zymo Research, Orange, CA, USA) following the manual. Purity and quality of the genomic DNA were checked on 0.8% agarose gels and NanoDrop 2000 spectrophotometer (Thermo Fisher Scientific).

PCR amplification. The V4 region of the 16S rRNA gene was amplified using primers 515F (GTGYCAGCMGCCGCGGTAA) and 806R (GGACTACHVGGGTWTCTAAT). PCR was performed using KOD-Plus-Neo DNA polymerase (TOYOBO) on an ABI 9700 system^25^. Each 50 μL reaction contained standard buffer, dNTPs, MgSO₄, primers (0.3 μM each), 1 U polymerase, and ~20 ng template DNA. Cycling conditions were: 94 °C for 1 min; 25–30 cycles of 94 °C for 20 s, 54 °C for 30 s, 72 °C for 30 s; and 72 °C for 5 min. Each sample was run in triplicate and pooled.

Amplicon processing and sequencing. PCR products were verified by 2% agarose gel electrophoresis, purified using Zymoclean Gel Recovery Kit, and quantified using a Qubit fluorometer. Equimolar amplicons were pooled and sequenced on an Illumina platform (PE250, HiSeq Rapid SBS Kit v2).

### 2.8 Bioinformatics and statistical analysis

Paired-end reads were merged using FLASH (v1.2.11) and demultiplexed using sabre. Quality filtering was performed in QIIME2 (v2020.2), removing reads with average quality score <30, length <200 bp, or ambiguous bases. Amplicon sequence variants (ASVs) were generated using the Deblur pipeline in QIIME2 (v2020.2). Taxonomy was assigned using a Naïve Bayes classifier trained on SILVA v138. Phylogenetic trees were constructed using FastTree. Samples were rarefied to equal sequencing depth prior to analysis. Alpha diversity (Chao1, Shannon, Simpson, Faith’s PD) was calculated in R (v4.0.5) using vegan and picante packages. Group differences were tested using Wilcoxon tests. Beta diversity was assessed using Bray–Curtis (Vegan package) and UniFrac distances (GUniFrac package). PCoA was performed in R (ape package, v4.0.5). Differential taxa were identified using random forest (randomForest package) performed in R (v4.0.5).

Statistical analyses were performed using IBM SPSS Statistics software (version 29.0; IBM Corp., Armonk, NY, USA). Each sheep was considered an experimental unit. Data were tested for normality using the Shapiro–Wilk test and for homogeneity of variance using Levene’s test. Except for the variables described above, all remaining data were analyzed by one-way ANOVA. When a significant treatment effect was detected, means were separated using Duncan’s multiple range test. For variables that did not meet the assumptions of normality, differences among treatments were evaluated using the Kruskal–Wallis test, followed by pairwise comparisons with adjustment for multiple testing. Pearson’s correlation analysis was conducted among the variables showing significant differences, and the correlation results were visualized using the online platform.^1^ Results are presented as means ± SEM. Statistical significance was declared at *p* < 0.05, and values of 0.05 ≤ *p* < 0.10 were considered to indicate a tendency toward significance.

## 3 Results

### 3.1 Trace minerals content in liver

The effect of CTM on the contents of TM in liver of growing sheep was shown in Table 2. The Fe, Cu, Mn, and Zn content in liver was increased by CTM compared to the ITM and CON group (*p* < 0.05), and the ITM was higher than the CON group (*p* < 0.05).

**Table 2:** Effects of coated trace minerals on the contents of trace minerals in liver of growing sheep/mg/kg.

Item	CON	ITM	CTM	SEM	*P*-Value
Fe	69.40^c^	91.21^b^	133.26^a^	8.18	<0.001
Cu	183.32^c^	233.42^b^	298.06^a^	15.90	0.001
Mn	10.34^c^	14.06^b^	16.65^a^	0.82	<0.001
Zn	74.66^c^	92.14^b^	98.53^a^	3.20	<0.001

CON, Control group; ITM, inorganic trace minerals group; CTM, coated trace minerals group; Fe, iron; Zn, zinc; Cu, copper; Mn, manganese. Values with different superscript letters differ significantly (*p* < 0.05).

### 3.2 Organ index

The effect of CTM on the organ index of growing sheep was shown in Table 3. The CTM increased the testis index of growing sheep compared to the CON and ITM group (*p* < 0.05). However, there was no difference in the organ index of heart, liver, lung, spleen, and kidney (*p* > 0.05).

**Table 3:** Effects of coated trace minerals on organ index of growing sheep/%.

Item	CON	ITM	CTM	SEM	*P*-Value
Heart	0.43	0.38	0.42	0.01	0.238
Hear	1.62	1.68	1.71	0.03	0.643
Lung	1.25	1.15	1.04	0.04	0.144
Spleen	0.14	0.11	0.12	0.01	0.243
Kidney	0.23	0.25	0.23	0.01	0.506
Testis	0.88^b^	0.94^b^	1.15^a^	0.05	0.032

CON, Control group; ITM, inorganic trace minerals group; CTM, coated trace minerals group. Values with different superscript letters differ significantly (*p* < 0.05).

### 3.3 Carcass characteristics

The effect of CTM on the slaughter performance of growing sheep was shown in Table 4. There was no difference in the live and carcass weight among all groups (*p* > 0.05), however, the ITM and CTM groups increased the slaughter rate (*p* < 0.05). For the half carcass weight, half bone weight, and half carcass meat bone ratio no differences between groups were observed (*p* > 0.05), but the half carcass meat rate had an increasing trend (0.05 < *p* < 0.1).

**Table 4:** Effects of coated trace minerals on slaughter performance of growing sheep.

Item	CON	ITM	CTM	SEM	*P*-Value
Live weight/kg	37.74	37.27	38.5	0.68	0.765
Carcass weight/kg	17.01	17.72	18.66	0.40	0.261
Slaughter rate/%	45.09^b^	47.46^a^	48.48^a^	0.54	0.011
Half carcass weight/kg	7.91	8.00	8.60	0.17	0.234
Half meat weight/kg	6.00	6.20	6.75	0.17	0.185
Half bone weight/kg	1.78	1.69	1.74	0.22	0.243
Half carcass meat rate/%	75.59	77.38	78.52	0.54	0.066
Half carcass meat bone ratio	3.38	3.68	3.90	0.11	0.117

CON, Control group; ITM, inorganic trace minerals group; CTM, coated trace minerals group. Values with different superscript letters differ significantly (*p* < 0.05).

### 3.4 Meat quality

The effect of CTM on the meat quality of growing sheep was shown in Table 5. The shear force of ITM and CTM group decreased compared to the CON (*p* < 0.05), and CTM group was lower compared to the ITM (*p* < 0.05). And there was an decreasing trend on the drip loss with the addition of trace elements (0.05 < *p* < 0.1). However, there was no effect on the pH_45min_, pH_24h_, cooking loss, L*_45min_, a*_45min_, and b*_45min_ (All *p* > 0.05).

**Table 5:** Effects of coated trace minerals on the meat quality of growing sheep.

Item	CON	ITM	CTM	SEM	*P*-Value
pH_45min_	6.41	6.59	6.36	0.06	0.217
pH_24h_	5.94	5.98	5.88	0.05	0.806
Shear force/*N*	72.85^a^	52.96^b^	43.12^c^	4.78	0.015
Cooking loss/%	39.94	38.79	36.84	1.10	0.555
Drip loss/%	3.71	2.64	1.84	0.34	0.065
L*_45min_	15.48	16.35	16.06	0.23	0.310
a*_45min_	6.69	6.84	7.01	0.18	0.799
b*_45min_	1.95	2.15	2.04	0.77	0.589

CON, Control group; ITM, inorganic trace minerals group; CTM, coated trace minerals group; a*, redness; b*, yellowness; L*, lightness. Values with different superscript letters differ significantly (*p* < 0.05).

### 3.5 Immunity and antioxidant performance

The effect of CTM on the serum immune and antioxidant performance of growing sheep was shown in Table 6. The immunoglobulin of A, G, and M were higher in the ITM and CTM group compared to the CON (*p* < 0.05), and CTM increased the serum concentration compared to the ITM (*p* < 0.05). For the C3 and C4, the CTM group concentration was the highest among all groups (*p* < 0.05), the ITM also increased the concentration of C3 and C4 compared to the CON (*p* < 0.05). Besides, the concentration of ET was decreased by the ITM and CTM (*p* < 0.05).

**Table 6:** Effects of coated trace minerals on serum immune and antioxidant performance of growing sheep.

Item	Item	CON	ITM	CTM	SEM	*p*-value
Immune performance	IgA (μg/mL)	30.20^c^	31.37^b^	32.54^a^	0.25	<0.001
	IgG (μg/mL)	1,162.44^c^	1,487.68^b^	1570.56^a^	31.30	<0.001
	IgM (μg/mL)	13.46^c^	13.86^b^	15.27^a^	0.15	<0.001
	C3 (μg/mL)	745.02^b^	760.89^b^	790.19^a^	5.45	0.001
	C4 (μg/mL)	238.61^c^	248.83^b^	260.91^a^	2.12	<0.001
	ET (EU/mL)	0.87^a^	0.82^b^	0.79^c^	0.01	<0.001
Antioxidant performance	T-SOD (U/mL)	98.59	99.79	97.42	0.54	0.209
	CAT (U/mL)	13.56^c^	24.75^b^	36.78^a^	3.21	0.001
	MDA (nmol/mL)	1.89^a^	1.42^b^	1.23^c^	0.09	<0.001

CON, Control group; ITM, inorganic trace minerals group; CTM, coated trace minerals group; IgA, immunoglobulin A; IgM, immunoglobulin M; IgG, immunoglobulin G; C3, complement C3; C4, complement C4; ET, endotoxin; SOD, superoxide dismutase; CAT, catalase; MDA, malondialdehyde. Values with different superscript letters differ significantly (*p* < 0.05).

The effect of CTM on the serum antioxidant performance of growing sheep was shown in Table 6. The CTM and ITM was increased the concentration of CAT and decreased the MDA concentration compared to the CON (*p* < 0.05). Moreover, the CAT was higher and the MDA was lower in the CTM compared to the ITM (*p* < 0.05).

### 3.6 Rumen fermentation

The effect of CTM on the rumen fermentation parameters of growing sheep was shown in Table 7. The pH had a decreasing trend with the ITM and CTM supplementation (0.05 < *p* < 0.1), and the NH_3_-N had an increasing trend (0.05 < *p* < 0.1). Besides, the MCP was higher in ITM and CTM group (*p* < 0.05). For the TVFA, the group of ITM and CTM were higher than the CON (*p* < 0.05), the acetic acid, butyrate acid, and isovaleric acid was also increased (*p* < 0.05). There was an increasing trend in propionic acid following ITM and CTM supplementation (0.05 < *p* < 0.1). However, no difference in the A/P was observed (*p* > 0.05), and none of the measured parameters differed significantly between the ITM and CTM groups (*p* > 0.05).

**Table 7:** Effects of coated trace minerals on rumen fermentation parameters of growing sheep.

Item	CON	ITM	CTM	SEM	*P*-Value
pH	6.91	6.61	6.66	0.61	0.080
NH_3_-N (mg/dL)	12.24	18.06	15.87	1.04	0.051
MCP (mg/mL)	1.73^b^	2.88^a^	2.66^a^	0.18	0.003
Acetic acid (mmol/L)	4.40^b^	6.43^a^	5.97^a^	0.34	0.019
Propionic acid (mmol/L)	1.20	1.82	1.47	0.12	0.074
Butyrate acid (mmol/L)	0.12^b^	0.19^a^	0.20^a^	0.01	0.008
Isobutyric acid (mmol/L)	0.24	0.21	0.24	0.02	0.840
Valeric acid (mmol/L)	0.15	0.26	0.18	0.02	0.141
Isovaleric acid (mmol/L)	0.07^b^	0.12^a^	0.11^a^	0.01	0.036
TVFA (mmol/L)	6.18^b^	9.04^a^	8.18^a^	0.48	0.024
A/P	3.73	3.56	4.17	0.15	0.246

CON, Control group; ITM, inorganic trace minerals group; CTM, coated trace minerals group; NH_3_-N, Ammonia nitrogen; MCP, microbial protein; TVFA, Total volatile fatty acids; A/P, acetic acid to propionic acid ratio. Values with different superscript letters differ significantly (*p* < 0.05).

### 3.7 Rumen bacterial structure and composition

**Figure 1:**
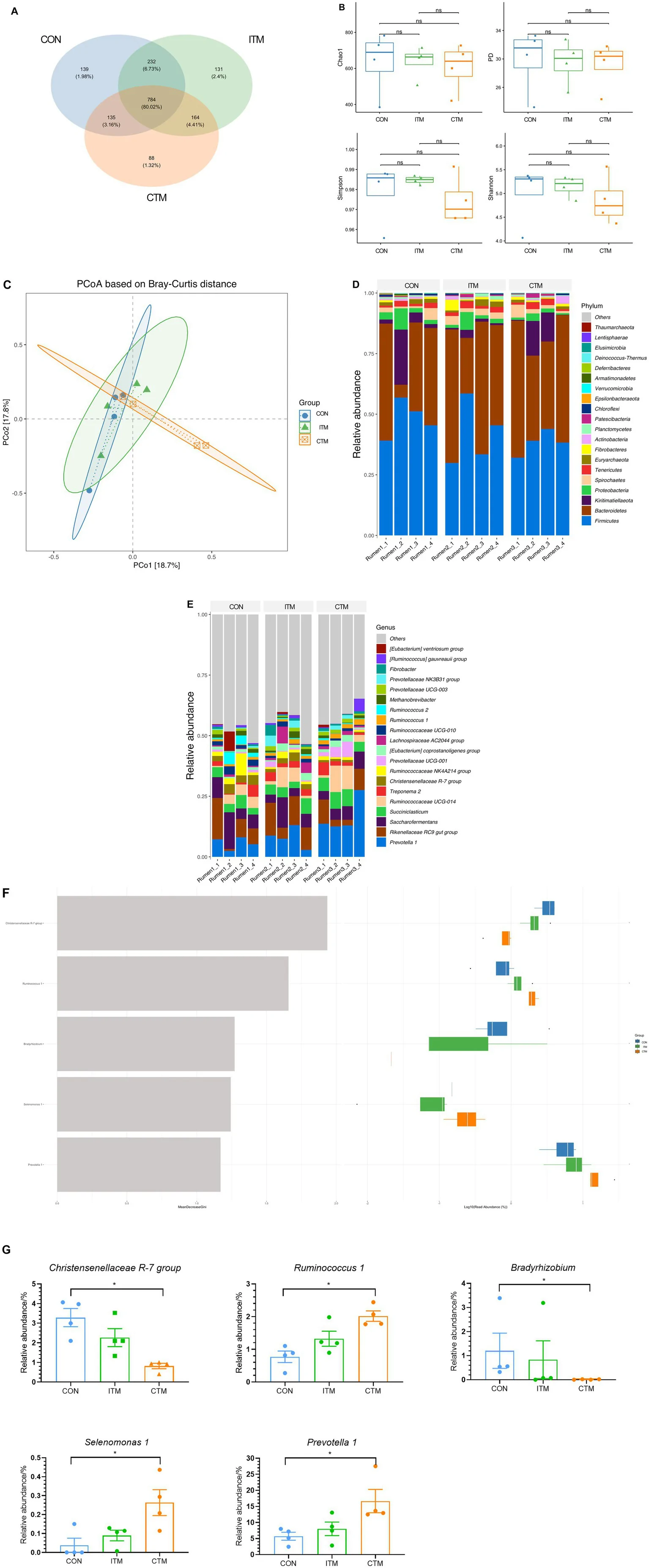
The effect of coated trace minerals on rumen bacterial structure and composition. **(A)** Venn diagram of amplicon sequence variants (ASVs) abundance. **(B)** Alpha diversity analysis of rumen bacteria. **(C)** Beta diversity analysis of rumen bacteria. The ellipses around each group represent the 95% confidence intervals. **(D)** Phylum-level bacterial composition in the rumen. **(E)** Genus-level bacterial composition in the rumen. **(F)** Identification of differential bacterial taxa using Random Forest and Kruskal–Wallis rank-sum test. **(G)** Analysis of differences in ruminal bacterial composition. *Differ significantly (*p* < 0.05). CON, Control group; ITM, inorganic trace minerals group; CTM, coated trace minerals group.

The effect of CTM on rumen bacterial structure and composition was shown in Figure 1. The rarefaction curves reached a plateau across all samples, indicating that the sequencing depth was sufficient to capture the majority of microbial diversity and that the sequencing data were reliable (Supplementary Figure 1). The ASVs abundance was shown in Venn diagram, the ASVs shared by all groups were 784, among which the CON, ITM, and CTM group were 139, 131, and 88, respectively (Figure 1A). The alpha-diversity was used to evaluate the species abundance or diversity in the rumen microbiome (Figure 1B), and no differences were observed on Chao 1, PD, Simpson, and Shannon in each treatment (All *p* > 0.05). The PCoA based on Bray–Curtis distance to evaluate Beta-diversity, the bacteria of CTM clustered separately from those of CON and ITM within a 95% confidence interval (Figure 1C).

The top 20 phylum and genus of rumen bacterial composition were shown in the Figures 1D,E. There were 22 and 226 taxonomically classified at phylum and genus level in the rumen microbiome. The dominant bacterial at phylum level were Firmicutes (38.32–48.13) and Bacteroidetes (32.60–45.11). The top 5 bacterial at genus level were *Prevotella* 1 (5.73–16.66), *Rikenellaceae RC9 gut group* (5.97–7.98), *Saccharofermentans* (5.20–8.44), *Succiniclasticum* (3.96–5.85), and *Ruminococcaceae UCG-014* (3.35–6.10).

We used a combination of Random Forest analysis and difference testing for analysis to identify key species with significant differences between groups (Figure 1F). Five differential bacteria were selected, including *Christensenellaceae R-7 group*, *Ruminococcus 1*, *Bradyrhizobium*, *Selenomonas 1*, and *Prevotella 1*. Compared with the CON group, CTM supplementation significantly increased the relative abundances of *Ruminococcus 1*, *Selenomonas 1*, and *Prevotella 1* (*p* < 0.05), whereas the relative abundances of *Christensenellaceae R-7* group and *Bradyrhizobium* were significantly reduced (*p* < 0.05) (Figure 1G).

### 3.8 Correlation analysis among multiple parameters

**Figure 2:**
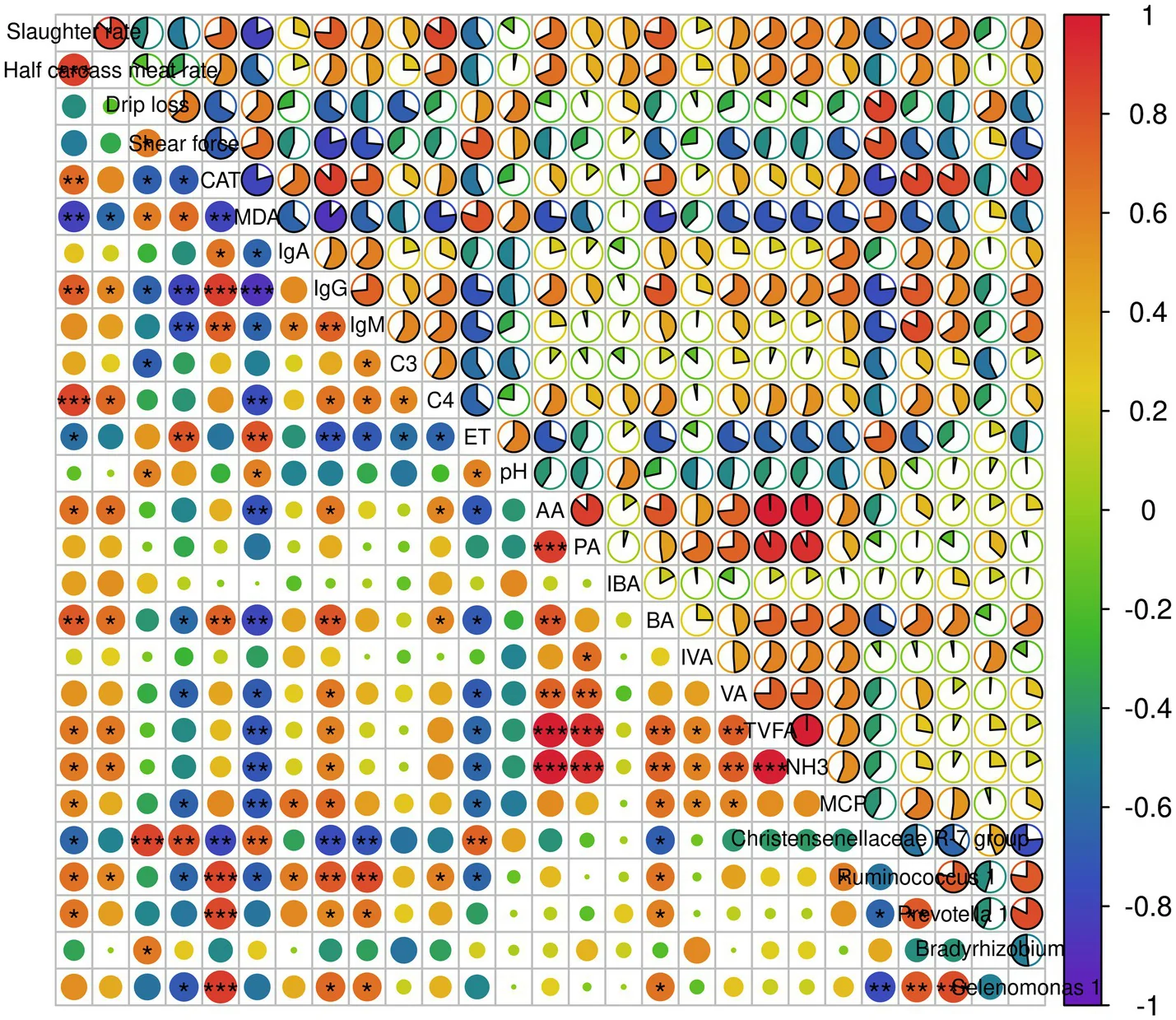
Correlation analysis among multiple parameters. Correlation coefficients were calculated using Pearson correlation analysis and are visualized using a color gradient ranging from −1 (blue, negative correlation) to 1 (red, positive correlation). Circle size (lower-left panel) and fill proportion (upper-right panel) represent the magnitude (absolute value) of correlation coefficients. Asterisks denote statistical significance (*p < 0.05, **p < 0.01, ***p < 0.001). CAT, catalase; MDA, malondialdehyde; IgA, immunoglobulin A; IgG, immunoglobulin G; IgM, immunoglobulin M; C3, complement component 3; C4, complement component 4; ET, endotoxin; AA, acetic acid; PA, propionic acid; IBA, isobutyric acid; BA, butyric acid; IVA, isovaleric acid; VA, valeric acid; TVFA, total volatile fatty acids; NH_3_, ammonia nitrogen; MCP, microbial protein.

Significant correlations among physiological and microbiome parameters were observed. Slaughter rate positively correlated with CAT, IgG, C4, AA, BA, MCP, TVFA, and NH₃, and negatively with MDA (*r* > 0.6, *p* < 0.05). Drip loss correlated positively with shear force, MDA, and pH, and negatively with CAT, IgG, and C3. Shear force positively correlated with MDA and ET, and negatively with CAT, IgG, IgM, BA, MCP, and VA (Figure 2).

Microbiota correlations showed *Prevotella 1* positively correlated with slaughter rate, CAT, IgG, IgM, BA. *Ruminococcus 1* positively correlated with slaughter rate, half carcass meat rate, CAT, IgA, IgG, IgM, C4, BA, and, MCP, and negatively with MDA, shear force, and ET. *Christensenellaceae R-7* positively correlated with shear force, drip loss, MDA, and, ET, and negatively with slaughter rate, CAT, IgM, IgG, and BA (*r* > 0.6, *p* < 0.05). *Bradyrhizobium* was positively correlated with drip loss (*r* > 0.6, *p* < 0.05). *Selenomonas 1* showed significant positive correlations with CAT, IgG, IgM, and BA, and a significant negative correlation with shear force (*r* > 0.6, *p* < 0.05).

At the level of taxa–taxa correlations, *Prevotella 1* was significantly positively correlated with *Ruminococcus 1* and *Selenomonas 1*, but negatively correlated with the *Christensenellaceae R-7 group* (*r* > 0.6, *p* < 0.05). In addition, *Christensenellaceae R-7 group* was significantly negatively correlated with *Selenomonas 1* (*r* > 0.6, *p* < 0.05).

## 4 Discussion

We systematically compared the effects of basal diet, ITM and CTM on immune performance, carcass characteristics, and meat quality of growing sheep. At the same time, the effects of TMs on rumen fermentation and rumen bacterial composition were expounded to reveal the mechanism of CTM and provide scientific basis for the application of TM. TM are essential for the optimal growth performance and health of fattening sheep. Mwangi et al. (2019) reported that the deposition of TM in tissues serves as an important indicator for evaluating the utilization, storage, and bioavailability of exogenous TM (Mwangi et al., 2019). The present results demonstrated that liver TM deposition in the CTMs group was greater than that in the ITM group, which in turn exceeded that of the CON group. These results are largely consistent with previous studies. For example, Wang et al. (2020) reported that hepatic Cu deposition in dairy cows supplemented with coated copper sulfate was 112.20% of that in cows receiving uncoated forms (Wang and Han et al., 2021a); Similarly, Wang et al. (2021b) found that liver Zn deposition in dairy cows supplemented with coated zinc sulfate reached 103.96% of that in the uncoated group. TM such as Cu, Fe, Mn, and Zn are essential for the growth and development of sheep and are primarily absorbed in the small intestine. The use of coating technology reduces the degradation and interaction of these TM by rumen microorganisms, thereby enhancing their availability for intestinal absorption.

Organ indices can, to some extent, reflect the developmental status of organs in animals. Although TM are present in relatively small quantities in the body, their roles in reproduction are critical and should not be overlooked. It has been reported that Zn is highly concentrated in the testes and accessory sex glands of bulls; thus, Zn deficiency can impair testicular development, reduce testicular weight, and ultimately compromise reproductive performance (Hidiroglou and Knipfel, 1984). Pitts et al. further demonstrated that feeding Zn-deficient diets to bulls for 13 weeks (from 8 to 21 weeks of age) resulted in reduced testicular volume compared with controls; however, when Zn supplementation was continued until 64 weeks of age, testicular volume recovered to normal levels (Pitts et al., 1966). Mn is also essential for the normal development of reproductive organs. Mn deficiency in bulls has been associated with reduced sperm production, diminished sexual function, and testicular degeneration, with severe cases leading to loss of reproductive capacity. Therefore, TM play a vital role in the reproductive performance of male livestock (Peng et al., 2025). In the present study, the testicular organ index in the CTM group was significantly higher than that in the other two groups. Combined with the observed liver TM concentrations, this result may be attributed to the enhanced liver deposition of CTM. Specifically, the liver concentrations of Zn and Mn in the CTM group were significantly higher than those in the other groups, suggesting that CTM may influence the development of male reproductive organs in sheep; however, further studies are required to verify its effects on reproductive function.

The level and form of TM supplementation can exert varying effects on carcass characteristics and meat quality in animals. Wu et al. reported that supplementation with mineral lick blocks increased carcass weight of Tibetan sheep by 11.18%, which is consistent with the findings of the present study (Wu et al., 2025). Compared with the CON group, supplementation with TM significantly improved slaughter rate in both the ITM and CTM groups by 5.26 and 7.52%, respectively. A similar increasing trend was also observed for half-carcass meat yield. Different supplementation forms may produce distinct effects; however, studies evaluating the effects of CTM on slaughter performance in ruminants remain limited. Yu (2017) reported that, in finishing pigs, dietary supplementation with CTM, compared with an equivalent level of ITM, increased slaughter rate by 3.19% and carcass length by 6.95 cm, indicating that coating enhances the beneficial effects of TM on slaughter performance (Yu, 2017). In contrast, Ke found no significant differences in slaughter performance between CTM and ITM in yellow-feathered broilers (Ke, 2015). The present results are consistent with the latter finding: although the slaughter rate in the CTM group (48.48%) was 1.02% higher than that in the ITM group (47.46%), the difference was not statistically significant. TM also play an important role in regulating meat quality, as both deficiency and excess may adversely affect product characteristics. Previous studies have shown that, in growing-finishing pigs, supplementation with CTM significantly reduced drip loss and shear force by 0.42 and 10.82%, respectively, compared with sulfate forms at equivalent dosages, while no significant differences were observed in cooking yield or meat color (Yu, 2017). Similarly, Yin et al. (2022a) reported that supplementation of 1,000 mg/kg CTM in broiler diets reduced drip loss and shear force of leg muscle by 0.21 and 8.95%, respectively. However, other studies have reported no significant effects of CTM on meat quality traits in poultry (Ke, 2015). In the present study, compared with the ITM group, the CTM group exhibited reductions of 30.3% in drip loss and 18.58% in shear force. These effects may be explained by several mechanisms. It has been suggested that, post-slaughter, muscle cell membranes are prone to disruption, leading to leakage of intracellular contents and increased water loss, thereby reducing nutritional value and storage stability (Malecki and Greger, 1996). In the present study, the CTM group exhibited greater antioxidant capacity than the ITM group, which likely enhanced free radical scavenging and improved water-holding capacity, thereby reducing drip loss. The lower shear force observed in the CTM group may be associated with increased liver TM deposition following CTM supplementation. First, coating facilitates greater delivery of TM to the intestine for absorption. Second, CTM may modulate rumen microbial populations, thereby improving nutrient digestion and utilization, enhancing TM absorption, and promoting intramuscular fat deposition. Collectively, these effects contribute to reduced shear force and improved meat tenderness.

Antioxidant capacity is considered a key indicator of animal growth performance and health status. MDA, a major product of lipid peroxidation, serves as an indicator of oxidative damage, whereas antioxidant enzymes such as T-SOD and CAT function as primary scavengers of reactive oxygen species (Yue et al., 2025; Zhao et al., 2022). Under physiological conditions, redox homeostasis is maintained in animals; however, this balance may be disrupted under specific conditions, such as environmental stress or impaired innate immunity, resulting in oxidative stress and subsequent deterioration of animal health. What’ s more, adequate dietary supplementation with TM is essential for maintaining the stability of the antioxidant defense system. Previous studies have demonstrated that OTM are more effective than inorganic forms in enhancing antioxidant capacity (Świątkiewicz et al., 2014). For instance, Liu (2019) reported that dietary supplementation with 250 mg/kg CTM in broilers significantly increased serum T-SOD, glutathione peroxidase, and total antioxidant capacity activities, while reducing MDA levels compared with uncoated forms (Liu, 2019). In the present study, dietary supplementation with different forms of TMs in sheep revealed that, compared with the ITM group, the CTM group exhibited a significant increase of 48.61% in serum CAT activity and a significant reduction of 13.38% in MDA content, indicating that CTM more effectively enhance antioxidant capacity in sheep. Combined with the observed hepatic TM deposition, improved absorption of Fe, Cu, Mn, and Zn can be inferred following CTM supplementation. Notably, Cu, Mn, and Zn are essential components of Cu/Zn-SOD and Mn-SOD; thus, their increased availability may enhance superoxide dismutase activity and thereby improve the scavenging of free radicals.

Immunoglobulins IgG, IgM, and IgA are widely recognized as key indicators of immune function and are closely associated with the immunocompetence of sheep. IgM, a fundamental antibody secreted by B lymphocytes, is the first immunoglobulin produced following antigen exposure and plays a critical role in early-stage immune responses, particularly in exocrine secretions. IgA exhibits antibacterial, antitoxic, and antiviral activities (Singh et al., 2014). IgG is the most abundant immunoglobulin in animal serum and constitutes a central component of systemic humoral immunity against infection. It is present at high concentrations, persists for extended periods, and contributes to bacterial inhibition, viral neutralization, and toxin neutralization (Li et al., 2025). However, limited information is available regarding the effects of CTM on serum immune parameters in animals. Zhu et al. (2015) reported that dietary supplementation with 2,400 mg/kg coated zinc oxide in weaned piglets significantly increased serum IgA, IgM, and IgG levels compared with equivalent doses of uncoated zinc oxide (Zhu et al., 2015). In contrast, Wang et al. (2020) reported that, compared with piglets fed 2,500 mg/kg uncoated zinc oxide, those receiving 500 mg/kg coated zinc oxide showed no significant differences in serum IgA and IgM levels, whereas IgG exhibited a slight but non-significant increasing trend (3.34%) (Wang et al., 2020). In the present study, compared with the ITM group, the CTM group showed significant increases in serum IgA, IgG, IgM, complement C3, and C4 levels, while ET levels were significantly reduced. These results are generally consistent with those of Zhu et al. (2015), but differ from those reported by Wang et al. (2020). This discrepancy may be attributed, in part, to the equal-dose experimental design employed in the present study, whereas Wang et al. (2020) applied a five-fold difference in supplementation levels between coated and uncoated treatments. In addition, variations in coating technology, coating materials, and animal species may also contribute to the inconsistent findings. Overall, these results suggest that, at equivalent supplementation levels, CTMs are more effective than inorganic forms in improving serum immune performance in animals.

Rumen fluid pH, VFA concentrations, and NH₃-N levels are key indicators for evaluating rumen fermentation function in ruminants and, to some extent, reflect the stability and normality of the ruminal environment (Zhao et al., 2025). In the present study, MCP production, as well as acetate, butyrate, and TVFA concentrations, were significantly increased in both the ITM and CTM groups compared with the CON group. In addition, rumen pH was reduced in the ITM group relative to the CON group. Spears (2003) reported that rumen degraded TM may influence fermentation processes through modula tion of microbial activity. Similarly, Pino and Heinrichs demonstrated that TM supplementation can enhance rumen fermentation (Pino and Heinrichs, 2016). This effect may be attributed to the regulatory role of CTM on rumen microbial activity, thereby promoting feed degradation and utilization and ultimately increasing VFA production. In our previous study, improved dietary protein utilization was observed following TM supplementation, which may also account for changes in NH₃-N concentrations (Zhou et al., 2022). Rumen NH₃-N levels have been reported to be closely associated with rumen-degradable protein breakdown and, to some extent, to reflect microbial activity (Agle et al., 2010). Consistently, MCP production was significantly higher in both the ITM and CTM groups than in the CON group in the present study. Rumen pH is a direct indicator of fermentation status, and a stable acid–base environment is essential for optimal rumen function, with a normal physiological range of 5.5–7.5 (Brown et al., 2006). In the present study, rumen pH remained within the normal range across all groups; however, both the ITM and CTM groups exhibited lower pH values than the CON group, likely due to increased VFA production. No significant differences in the A/P were observed among groups, which may be associated with interactions among TM in the rumen. Several studies have suggested that the form of TM (coated vs. uncoated) has limited effects on rumen fermentation characteristics. Wang et al. (2020) reported that, in dairy cows supplemented with coated copper sulfate at equivalent levels, no significant differences were observed in pH, acetate, propionate, butyrate, TVFA, A/P, or NH₃-N compared with uncoated forms (Wang and Han et al., 2021a). Similarly, Zhang et al. (2020) reported comparable findings in studies involving coated sodium selenite (Zhang et al., 2020). In the present study, no significant differences in rumen fermentation parameters were observed between the CTM and ITM groups. This may be explained by the ruminal bypass rate of CTM being 65.87%, suggesting that approximately 34% of supplemented trace minerals were still released within the rumen. These findings suggest that CTM may allow a greater proportion of TM to bypass the rumen for intestinal absorption, without adversely affecting rumen fermentation. In summary, the form of TMs supplementation did not significantly alter rumen fermentation characteristics in sheep. However, TM supplementation per se promoted rumen fermentation, which may contribute to rumen homeostasis maintenance and improved production performance.

Rumen microorganisms play a critical role in maintaining rumen homeostasis and supporting host metabolic function. However, limited information is currently available regarding the effects of CTM on rumen fermentation and microbial community structure. In terms of *α*-diversity, no marked differences were observed among the CON, ITM, and CTM groups, with 80.02% of ASVs shared across all treatments. To identify key taxa that differed significantly among treatments, a Random Forest analysis combined with differential abundance testing was performed. This integrated approach enables the identification of key differential bacteria by ranking taxa according to their importance in group classification, thereby facilitating interpretation when combined with relative abundance and other biological information. At the genus level, five key differential bacteria were identified, including *Christensenellaceae R-7 group*, *Ruminococcus 1*, *Prevotella 1*, *Bradyrhizobium*, and *Selenomonas 1*. Further differential analysis revealed that *Ruminococcus 1*, *Prevotella 1*, and *Selenomonas 1* were most abundant in the CTM group, followed by the ITM group, with the lowest abundance observed in the CON group, whereas the *Christensenellaceae R-7 group* exhibited the opposite pattern. Previous studies have reported that OTM supplementation, compared with inorganic forms, can increase the abundance of *Prevotella bryantii*. *Prevotella 1* is a dominant rumen genus capable of producing a wide range of extracellular enzymes that degrade complex carbohydrates, proteins, and amino acids, thereby providing substrates for lipid, protein, and carbohydrate metabolism (Grabner et al., 2023; Kljak et al., 2017). Although *Prevotella* spp. do not directly degrade fiber, co-culture studies have shown that they can enhance the activity of cellulolytic bacteria and are closely associated with improved dietary fiber digestibility. Consistent with these findings, our previous study demonstrated that CTM supplementation improved apparent crude protein digestibility compared with ITM (Zhou et al., 2022). In addition, microbial enterotypes dominated by *Succinivibrio* and members of the *Prevotellaceae* family have been reported to be enriched in amino acid biosynthesis pathways, further supporting the present findings (Wang et al., 2023). Correlation analysis in this study further showed that *Prevotella 1* was significantly positively associated with slaughter rate, CAT activity, and butyrate acid. Recent evidence also suggests that ruminal *Prevotella* spp. may enhance glutathione and VFA metabolism, thereby promoting adenosine triphosphate and nucleotide synthesis (Mao et al., 2026). Collectively, these results indicate that *Prevotella 1* may associated with improved antioxidant capacity and enhanced energy metabolism in the host. *Ruminococcus 1* is also a well-established fibrolytic bacterium capable of degrading cellulose and hemicellulose (Liu et al., 2026). Previous studies have suggested that members of the *Ruminococcaceae* family may influence fat deposition in mice (Zhao et al., 2017). In the present study, *Ruminococcus 1* showed significant positive correlations with slaughter rate, half carcass meat rate, CAT, IgA, IgG, IgM, C4, BA, and, and *Prevotella 1*, and significant negative correlations with MDA, shear force, ET. Recent research has also reported a strong positive association between *Ruminococcus 1* abundance and marbling scores in sheep, suggesting a potential role in intramuscular fat deposition (Yang et al., 2026). Moreover, *Ruminococcaceae* UCG-004 has been associated with higher levels of unsaturated fatty acids, including oleic acid, linolenic acid, eicosapentaenoic acid, and docosahexaenoic acid (Xiang et al., 2022). The *Christensenellaceae* family is recognized as one of the most heritable microbial taxa in the human gut microbiome and is negatively associated with host body weight (Goodrich et al., 2014). In pigs, the *Christensenellaceae R-7 group* has been reported to be negatively correlated with the ratio of goblet cells to villus height (McCormack et al., 2017). Similarly, in yaks, a higher abundance of the *Christensenellaceae R-7 group* has been observed in growth-retarded individuals compared with normally growing animals (Ma et al., 2020). In the present study, the relative abundance of the *Christensenellaceae R-7 group* was lowest in the CTM group. Correlation analysis further indicated that *Christensenellaceae R-7 group* negatively with slaughter rate, CAT, IgM, IgG, and BA, and positively correlated with shear force, drip loss, MDA, and, ET. Meanwhile, *Selenomonas-1* showed the highest relative abundance in the CTMs group, and a significant negative correlation with shear force was observed. This may be attributed to the ability of certain *Selenomonas* species (e.g., *Selenomonas ruminantium*) to produce short-chain fatty acids, such as acetate and propionate, from fermentation intermediates including lactate and succinate, which can subsequently be utilized by the host as energy substrates (Holman et al., 2024). Importantly, the genus *Selenomonas* has been associated with increased marbling in Angus steers (Krause et al., 2020) and improved feed efficiency in dairy cattle (Xue et al., 2022). Moreover, in the present study, significant correlations among these bacterial taxa were observed. *Prevotella 1* showed positive correlations with *Ruminococcus 1* and *Selenomonas 1*, whereas it was negatively correlated with the *Christensenellaceae R-7 group*. In addition, the *Christensenellaceae R-7 group* exhibited a negative correlation with *Selenomonas 1*. Previous studies have also reported a negative relationship between the *Christensenellaceae R-7 group* and members of the *Prevotellaceae* family (Wang et al., 2023), which may be explained by ecological interactions within microbial communities, where taxa occupying similar ecological niches compete for limited nutrients and space, leading to mutual exclusion. The patterns of microbial coexistence and exclusion are largely driven by evolutionary relatedness and functional similarity (Faust and Raes, 2012; Faust et al., 2012). In addition, studies on CTM have shown that they can modulate intestinal microbiota, upregulate carbohydrate metabolism pathways, and stimulate glycerolipid metabolism and membrane transport, thereby improving growth performance in ducks (Yin et al., 2022b). Yin et al. (2022) also demonstrated that CTM improve lipid metabo lism in broilers (Yin et al., 2022a). In summary, the present results provide a comprehensive characterization of the effects of CTM on rumen microbial structure and identify key fibrolytic bacterial taxa. *Christensenellaceae R-7 group*, *Ruminococcus 1*, *Selenomonas 1*, and *Prevotella 1* were all closely associated with fiber degradation, which may have important implications for sheep health and growth performance.

Several limitations of the present study should be considered. The relatively short feeding period and limited number of animals may have constrained the assessment of the long-term effects of CTM supplementation on carcass characteristics, meat quality, and rumen microbiota development. Therefore, future investigations with larger sample sizes, longer supplementation periods, and comprehensive functional analyses are needed to further elucidate the mechanisms underlying the beneficial effects of CTM in sheep.

## 5 Conclusion

This study provides further evidence that dietary supplementation with 400 mg/kg coated trace minerals (CTM) can modulate the rumen microbial community, as reflected by changes in the relative abundances of bacterial taxa associated with fiber degradation, including *Ruminococcus 1*, *Prevotella 1*, *Selenomonas 1*, and the *Christensenellaceae R-7 group*. Compared with inorganic trace minerals (ITM), CTM supplementation resulted in greater hepatic mineral deposition, improved serum immune parameters, and enhanced antioxidant capacity. Additionally, CTM reduced shear force compared with both ITM and the control, suggesting improved meat tenderness. While slaughter rate was improved by both ITM and CTM relative to the control, no significant difference was observed between the two supplemented groups. Furthermore, correlated analysis suggested that CTM-induced microbiota modulation may be linked to improved host phenotypic outcomes. Collectively, these findings provide evidence supporting the application of CTM as an effective nutritional strategy to improve carcass characteristics, meat quality, and health in sheep.

## Data Availability

The raw microbial sequence data reported in this article have been deposited in the NCBI Sequence Read Archive (SRA) under BioProject accession PRJNA1498727 (https://www.ncbi.nlm.nih.gov/sra/PRJNA1498727).

## Footnotes

1
https://www.omicstudio.cn/tool/140

## References

[ref1] Agle M. Hristov A. N. Zaman S. Schneider C. Ndegwa P. Vaddella V. K. (2010). The effects of ruminally degraded protein on rumen fermentation and ammonia losses from manure in dairy cows. J. Dairy Sci. 93, 1625–1637. doi: 10.3168/jds.2009-2579, 20338440

[ref2] Andrieu S. (2008). Is there a role for organic trace element supplements in transition cow health? Vet. J. 176, 77–83. doi: 10.1016/j.tvjl.2007.12.022, 18329303

[ref3] Berthiaume R. Benchaar C. Chaves A. V. Tremblay G. F. Castonguay Y. Bertrand A. . (2010). Effects of nonstructural carbohydrate concentration in alfalfa on fermentation and microbial protein synthesis in continuous culture. J. Dairy Sci. 93, 693–700. doi: 10.3168/jds.2009-2399, 20105540

[ref4] Brady T. J. Feuz R. Reichhardt C. C. Motsinger L. A. Ineck N. E. Briggs R. K. . (2025). The effects of different trace mineral supplementation strategies on performance, feeding behavior, health, carcass quality, and profitability of mineral deficient receiving steers. Domest. Anim. Endocrinol. 95:106981. doi: 10.1016/j.domaniend.2025.106981, 41353984

[ref5] Brown M. S. Ponce C. H. Pulikanti R. (2006). Adaptation of beef cattle to high-concentrate diets: performance and ruminal metabolism. J. Anim. Sci. 84, E25–E33. doi: 10.2527/2006.8413_supplE25x 16582090

[ref6] Brugger D. Wagner B. Windisch W. M. Schenkel H. Schulz K. Südekum K. . (2022). Bioavailability of trace elements in farm animals: definition and practical considerations for improved assessment of efficacy and safety. Animal 16:100598. doi: 10.1016/j.animal.2022.100598 35952480

[ref7] Durand M. Kawashima R. (1980). Influence of Minerals in rumen Microbial Digestion. Paper Presented at the Digestive Physiology and Metabolism in Ruminants: Proceedings of the 5th International Symposium on Ruminant Physiology, held at Clermont—Ferrand, on 3rd–7th September, 1979. Berlin: Springer.

[ref8] Erwin E. S. Marco G. J. Emery E. M. (1961). Volatile fatty acid analyses of blood and rumen fluid by gas chromatography. J. Dairy Sci. 44, 1768–1771. doi: 10.3168/jds.S0022-0302(61)89956-6

[ref9] Faust K. Raes J. (2012). Microbial interactions: from networks to models. Nat. Rev. Microbiol. 10, 538–550. doi: 10.1038/nrmicro2832, 22796884

[ref10] Faust K. Sathirapongsasuti J. F. Izard J. Segata N. Gevers D. Raes J. . (2012). Microbial co-occurrence relationships in the human microbiome. PLoS Comput. Biol. 8:e1002606. doi: 10.1371/journal.pcbi.1002606, 22807668 PMC3395616

[ref11] Goff J. P. (2018). Invited review: mineral absorption mechanisms, mineral interactions that affect acid–base and antioxidant status, and diet considerations to improve mineral status. J. Dairy Sci. 101, 2763–2813. doi: 10.3168/jds.2017-13112, 29397180

[ref12] Goodrich J. K. Waters J. L. Poole A. C. Sutter J. L. Koren O. Blekhman R. . (2014). Human genetics shape the gut microbiome. Cell 159, 789–799. doi: 10.1016/j.cell.2014.09.053, 25417156 PMC4255478

[ref13] Grabner E. Stare E. Fanedl L. Zorec M. Jones D. S. Johnston C. D. . (2023). Expanding the rumen *Prevotella* collection: the description of *Prevotella communis*, sp. Nov. of ovine origin. Syst. Appl. Microbiol. 46:126437. doi: 10.1016/j.syapm.2023.126437, 37295348 PMC11198866

[ref14] Hidiroglou M. Knipfel J. E. (1984). Zinc in mammalian sperm: a review. J. Dairy Sci. 67, 1147–1156. doi: 10.3168/jds.S0022-0302(84)81416-2, 6378991

[ref15] Holman D. B. Gzyl K. E. Scott H. Service, C Prieto N. López-Campos Ó. (2024). Associations between the rumen microbiota and carcass merit and meat quality in beef cattle. Appl. Microbiol. Biotechnol. 108:287. 38581592 10.1007/s00253-024-13126-1 PMC10998782

[ref16] Hurlbert J. L. Menezes A. C. B. Baumgaertner F. Bochantin-Winders K. A. Jurgens I. M. Kirsch J. D. . (2024). Vitamin and mineral supplementation to beef heifers during gestation: impacts on morphometric measurements of the neonatal calf, vitamin and trace mineral status, blood metabolite and endocrine profiles, and calf organ characteristics at 30 h after birth. J. Anim. Sci. 102:skae116. doi: 10.1093/jas/skae116, 38666437 PMC11121445

[ref17] Ke F. (2015). Study on Application of Micro-Coating Slow-Releasing Compound Trace Mineral on Broilers. Fuzhou: Fujian Agriculture and Forestry University.

[ref18] Kljak K. Pino F. Harvatine K. J. Heinrichs A. J. (2017). Analysis of selected rumen microbial populations in dairy heifers limit fed diets varying in trace mineral form and starch content. Livest. Sci. 198, 93–96. doi: 10.1016/j.livsci.2017.02.012

[ref19] Krause T. R. Lourenco J. M. Welch C. B. Rothrock M. J. Callaway T. R. Pringle T. D. (2020). The relationship between the rumen microbiome and carcass merit in Angus steers. J. Anim. Sci. 98:skaa287. doi: 10.1093/jas/skaa287, 32877916 PMC7526868

[ref20] Li X. An N. Chen H. Liu D. (2025). Effects of yeast culture on growth performance, antioxidant capacity, immune function, and intestinal microbiota structure in Simmental beef cattle. Front. Vet. Sci. 11:1533081. doi: 10.3389/fvets.2024.1533081, 39959843 PMC11827572

[ref21] Li X. Wang Y. Xu J. Yang Q. Sha Y. Jiao T. . (2024). Effects of yeast cultures on meat quality, flavor composition and rumen microbiota in lambs. Curr. Res. Food Sci. 9:100845. doi: 10.1016/j.crfs.2024.100845, 39376582 PMC11456904

[ref22] Liu S. (2019). Effects of Coated trace Minerals on Theperformance and Health of Broilers. Ya'an: Sichuan Agricultural University.

[ref23] Liu M. Su N. Ma Z. Chen W. Zhang Y. Yan X. . (2026). Meat quality differences correlated with rumen microbiota and lipid metabolism in beef cattle vs. castrated cattle. Int. J. Mol. Sci. 27:2296. doi: 10.3390/ijms27052296, 41828516 PMC12986454

[ref24] Liu B. Xiong P. Chen N. He J. Lin G. Xue Y. . (2016). Effects of replacing of inorganic trace minerals by organically bound trace minerals on growth performance, tissue mineral status, and fecal mineral excretion in commercial grower-finisher pigs. Biol. Trace Elem. Res. 173, 316–324. doi: 10.1007/s12011-016-0658-7, 26920735

[ref25] Lu W. B. Kuang Y. G. Ma Z. X. Liu Y. G. (2020). The effect of feeding broiler with inorganic, organic, and coated trace minerals on performance, economics, and retention of copper and zinc. J. Appl. Poult. Res. 29, 1084–1090. doi: 10.1016/j.japr.2020.10.002

[ref26] Ma J. Zhu Y. Wang Z. Yu X. Hu R. Wang X. . (2020). Comparing the bacterial community in the gastrointestinal tracts between growth-retarded and normal yaks on the Qinghai–Tibetan plateau. Front. Microbiol. 11:600516. doi: 10.3389/fmicb.2020.600516, 33391217 PMC7775487

[ref27] Malecki E. A. Greger J. L. (1996). Manganese protects against heart mitochondrial lipid peroxidation in rats fed high levels of polyunsaturated fatty acids. J. Nutr. 126, 27–33. doi: 10.1093/jn/126.1.27, 8558311

[ref28] Mao K. Zang Y. Wang C. Yang W. Lu G. Qiu Q. . (2026). Rumen microbiota-associated stress alleviation by creatine pyruvate in newly received cattle: a multi-omics study. Microbiome 14:114. doi: 10.1186/s40168-026-02365-1, 41782139 PMC13069763

[ref29] McCormack U. M. Curião T. Buzoianu S. G. Prieto M. L. Ryan T. Varley P. . (2017). Exploring a possible link between the intestinal microbiota and feed efficiency in pigs. Appl. Environ. Microbiol. 83, e00317–e00380. doi: 10.1128/AEM.00380-17, 28526795 PMC5514681

[ref30] Mion B. Van Winters B. King K. Spricigo J. Ogilvie L. Guan L. . (2022). Effects of replacing inorganic salts of trace minerals with organic trace minerals in pre-and postpartum diets on feeding behavior, rumen fermentation, and performance of dairy cows. J. Dairy Sci. 105, 6693–6709. 35787325 10.3168/jds.2022-21908

[ref31] Mwangi S. Timmons J. Ao T. Paul M. Macalintal L. Pescatore A. . (2019). Effect of manganese preconditioning and replacing inorganic manganese with organic manganese on performance of male broiler chicks. Poult. Sci. 98, 2105–2113. doi: 10.3382/ps/pey564, 30590788 PMC6448132

[ref37] NASEM (The National Academies of Sciences, Engineering, and Medicine). (2016). Nutrient Requirements of Beef Cattle. 8th ed.; Washington, DC, USA: National Academies Press.

[ref32] Peng D. Feng F. Yin H. Zhao J. Cao S. Liu J. (2025). Manganese deficiency causes testicular developmental disorders, blood–testis barrier damage, and spermatogenesis disruption via Nrf2-mediated oxidative stress. Nutrients 17:3007. doi: 10.3390/nu17183007, 41010532 PMC12472890

[ref33] Pino F. Heinrichs A. J. (2016). Effect of trace minerals and starch on digestibility and rumen fermentation in diets for dairy heifers. J. Dairy Sci. 99, 2797–2810. doi: 10.3168/jds.2015-10034, 26851846

[ref34] Pitts W. J. Miller W. J. Fosgate O. T. Morton J. D. Clifton C. M. (1966). Effect of zinc deficiency and restricted feeding from two to five months of age on reproduction in Holstein bulls. J. Dairy Sci. 49, 995–1000. doi: 10.3168/jds.S0022-0302(66)87997-3, 5969001

[ref35] Ren Y. Xue B. Hong Q. Kuang Y. Zhou Y. Shao L. . (2023). Effects of coated compound trace elements on growth performance, serum antioxidant and immuneindices of Yunnan semi-fine-wool sheep during growing period. Heilongjiang Anim Sci Vet Med 11, 98–105. doi: 10.13881/j.cnki.hljxmsy.2022.06.0210

[ref36] Santos T. Connolly C. Murphy R. (2015). Trace element inhibition of phytase activity. Biol. Trace Elem. Res. 163, 255–265. doi: 10.1007/s12011-014-0161-y, 25416530

[ref38] Singh K. Chang C. Gershwin M. E. (2014). IgA deficiency and autoimmunity. Autoimmun. Rev. 13, 163–177. doi: 10.1016/j.autrev.2013.10.005, 24157629

[ref39] Spears J. W. (2003). Trace mineral bioavailability in ruminants. J. Nutr. 133, 1506S–1509S. doi: 10.1093/jn/133.5.1506S, 12730454

[ref40] Spears J. W. Brandao V. Heldt J. (2022). Invited review: assessing trace mineral status in ruminants, and factors that affect measurements of trace mineral status. Appl. Anim. Sci. 38, 252–267. doi: 10.15232/aas.2021-02232

[ref41] Świątkiewicz S. Arczewska-Włosek A. Jozefiak D. (2014). The efficacy of organic minerals in poultry nutrition: review andimplications of recent studies. Worlds Poult. Sci. J. 70, 475–486. doi: 10.1017/s0043933914000531

[ref42] Van Emon M. Sanford C. McCoski S. (2020). Impacts of bovine trace mineral supplementation on maternal and offspring production and health. Animals 10:2404. doi: 10.3390/ani10122404, 33339123 PMC7765511

[ref43] Vigh A. Criste A. D. Corcionivoschi N. Gerard C. (2023). Rumen Solubility of Copper, Manganese and Zinc and the Potential Link between the Source and Rumen Function: A Systematic Review. Agriculture, 13:2198. doi: 10.3390/agriculture13122198

[ref44] Wang Y. Chen H. Wan M. Chen Y. Wang F. Zhu A. . (2020). Effects of coated nano-zinc oxide on growth performance, activity of antioxidation enzyme and serum biochemical, immune index of weaned piglets. Chin. J. Anim. Sci. 56, 117–121. doi: 10.19556/j.0258-7033.20190425-08

[ref45] Wang C. Han L. Zhang G. W. Du H. S. Wu Z. Z. Liu Q. . (2021). Effects of copper sulphate and coated copper sulphate addition on lactation performance, nutrient digestibility, ruminal fermentation and blood metabolites in dairy cows. Br. J. Nutr. 125, 251–259. doi: 10.1017/S0007114520002986, 32718368

[ref46] Wang D. Tang G. Wang Y. Yu J. Chen L. Chen J. . (2023). Rumen bacterial cluster identification and its influence on rumen metabolites and growth performance of young goats. Anim. Nutr. 15, 34–44. doi: 10.1016/j.aninu.2023.05.013, 37771855 PMC10522951

[ref47] Wang D. Tang G. Yu J. Li Y. Feng L. Liu H. . (2023). Microbial enterotypes shape the divergence in gut fermentation, host metabolism, and growth rate of young goats. Microbiol. Spectrum 11, e04818–e04822. doi: 10.1128/spectrum.04818-22, 36625605 PMC9927581

[ref48] Wang C. Xu Y. Z. Han L. Liu Q. Guo G. Huo W. J. . (2021b). Effects of zinc sulfate and coated zinc sulfate on lactation performance, nutrient digestion and rumen fermentation in Holstein dairy cows. Livest. Sci. 251:104673. doi: 10.1016/j.livsci.2021.104673

[ref49] Weatherburn M. W. (1967). Phenol-hypochlorite reaction for determination of ammonia. Anal. Chem. 39, 971–974. doi: 10.1021/ac60252a045

[ref50] Wu G. Zhao J. Zhao X. Wang L. Bai B. Yang Y. . (2025). Effects of supplementary feeding mineral licking brick on growth performance, slaughter performance, rumen fermentation parameters and microbial community, and serum indexes of Tibetan sheep. Chin. J. Anim. Nutr. 37, 4713–4727. doi: 10.12418/CJAN2025.385

[ref51] Xiang J. Zhong L. Luo H. Meng L. Dong Y. Qi Z. . (2022). A comparative analysis of carcass and meat traits, and rumen bacteria between Chinese Mongolian sheep and dorper× Chinese Mongolian crossbred sheep. Animal 16:100503. doi: 10.1016/j.animal.2022.100503, 35378496

[ref52] Xue M. Xie Y. Zhong Y. Ma X. Sun H. Liu J. (2022). Integrated meta-omics reveals new ruminal microbial features associated with feed efficiency in dairy cattle. Microbiome 10:32. doi: 10.1186/s40168-022-01228-9, 35172905 PMC8849036

[ref53] Yang Y. Luo X. Lu D. Du P. Jier S. Wu X. . (2026). Meat quality differences between Ganan Tibetan sheep and Tianzhu Tibetan sheep using metabolomics and rumen microbiota analyses. Microorganisms. 14:575. doi: 10.3390/microorganisms14030575, 41900337 PMC13028713

[ref54] Yin D. Tong T. Moss A. F. Zhang R. Kuang Y. Zhang Y. . (2022a). Effects of coated trace minerals and the fat source on growth performance, antioxidant status, and meat quality in broiler chickens. J. Poult. Sci. 59, 56–63. doi: 10.2141/jpsa.0200108, 35125913 PMC8791779

[ref55] Yin D. Zhai F. Lu W. Moss A. F. Kuang Y. Li F. . (2022b). Comparison of coated and uncoated trace minerals on growth performance, tissue mineral deposition, and intestinal microbiota in ducks. Front. Microbiol. 13:831945. doi: 10.3389/fmicb.2022.831945, 35495727 PMC9039745

[ref56] Yu Z. (2017). Study on the Effects of Coated Trace Elements in the Growing-Finishing Pigs. Master, Shenyang Agricultural University

[ref57] Yue Z. Ke S. Shi L. Hu R. Wang Z. Peng Q. . (2025). Dietaryv glutamine supplementation alleviated rumen epithelium oxidative damage and apoptosis induced by feed restriction via maintaining mitochondrial homeostasis in female yaks. Anim. Res. One Health. 1–11. doi: 10.1002/aro2.70048

[ref58] Zhang Z. D. Wang C. Du H. S. Liu Q. Guo G. Huo W. J. . (2020). Effects of sodium selenite and coated sodium selenite on lactation performance, total tract nutrient digestion and rumen fermentation in Holstein dairy cows. Animal 14, 2091–2099. doi: 10.1017/S1751731120000804, 32340650

[ref59] Zhang W. Wang M. Liu C. Li C. He X. Wang H. . (2026). Rumen microbiota–host interactions regulate intramuscular fat deposition in cattle via the alpha-linolenic acid–fatty acid β-oxidation/L-carnitine–MPO axis. Microbiome 14:49. doi: 10.1186/s40168-025-02252-1, 41372768 PMC12853858

[ref60] Zhao S. Shan C. Wu Z. Feng M. Song L. Wang Y. . (2022). Fermented Chinese herbal preparation: impacts on milk production, nutrient digestibility, blood biochemistry, and antioxidant capacity of late-lactation cows under heat stress. Anim. Feed Sci. Technol. 292:115448. doi: 10.1016/j.anifeedsci.2022.115448

[ref61] Zhao S. Sun X. Zhao N. Zhang H. Wang G. Zhang L. . (2025). Fermented Chinese herbal medicines combined with probiotics promote growth of fattening lambs under heat stress by modulating rumen mucosal barrier functions. Anim. Feed Sci. Technol. 326:116384. doi: 10.1016/j.anifeedsci.2025.116384

[ref62] Zhao L. Zhang Q. Ma W. Tian F. Shen H. Zhou M. (2017). A combination of quercetin and resveratrol reduces obesity in high-fat diet-fed rats by modulation of gut microbiota. Food Funct. 8, 4644–4656. doi: 10.1039/C7FO01383C, 29152632

[ref63] Zhou J. Ren Y. Wen X. Yue S. Wang Z. Wang L. . (2022). Comparison of coated and uncoated trace elements on growth performance, apparent digestibility, intestinal development and microbial diversity in growing sheep. Front. Microbiol. 13:1080182. doi: 10.3389/fmicb.2022.1080182, 36605519 PMC9808050

[ref64] Zhu Y. Su X. Li F. Zhang X. Kuang Y. Zheng L. . (2015). Effects of coated ZnO on growth performance, serum biochemical indices and nutrient apparent digestibility of weaner piglets. Chin. J. Anim. Nutr. 27, 1779–1786. doi: 10.3969/j.issn.1006-267x.2015.06.014

